# Postoperative Atrial Fibrillation Prediction by Left Atrial Size in Coronary Artery Bypass Grafting and Five-Year Survival Outcome

**DOI:** 10.3390/jcm13133738

**Published:** 2024-06-26

**Authors:** Mustafa Gerçek, Jochen Börgermann, Jan Gummert, Muhammed Gerçek

**Affiliations:** 1 Heart Center Duisburg, Clinic for Cardiac Surgery and Pediatric Cardiac Surgery, Gerrickstraße 21, 47137 Duisburg, Germany; mustafa.gercek@evkln.de (M.G.); jochen.boergermann@evkln.de (J.B.); 2 Herz- und Diabeteszentrum NRW, Clinic for Thoracic and Cardiovascular Surgery, NRW, Ruhr-Universität Bochum, Medizinische Fakultät OWL (Universität Bielefeld), Georgstraße 11, 32545 Bad Oeynhausen, Germany; jgummert@hdz-nrw.de; 3 Herz- und Diabeteszentrum NRW, Clinic for General and Interventional Cardiology/Angiology, NRW, Ruhr-Universität Bochum, Medizinische Fakultät OWL (Universität Bielefeld), Georgstraße 11, 32545 Bad Oeynhausen, Germany

**Keywords:** POAF, LAA amputation, cardiac surgery, OPCAB, off-pump

## Abstract

**Background**: Postoperative Atrial Fibrillation (POAF) is a common complication in cardiac surgery. Despite its multifactorial origin, the left atrial (LA) size is closely linked to POAF, raising the question of a valid cut-off value and its impact on the long-term outcome. **Methods**: Patients without a history of AF who underwent coronary artery bypass grafting between 2014 and 2016 were selected for this retrospective study. LA size was preoperatively assessed using the left atrial anterior–posterior diameter (LAAPd). Correlation and logistic regression analyses were performed, following a receiver-operating characteristic (ROC) analysis. Propensity score matching (PSM) was applied to ensure group comparability, followed by a comparison analysis regarding the primary endpoint of POAF and the secondary endpoints of all-cause mortality and stroke during a five-year follow-up. **Results**: A total of 933 patients were enrolled in the study eventually revealing a significant correlation between LAAPd and POAF (cor = 0.09, *p* < 0.01). A cut-off point of 38.5 mm was identified, resulting in groups with 366 patients each after PSM. Overall, patients with a dilated LA presented a significantly higher rate of POAF (22.3% vs. 30.4%, *p* = 0.02). In a five-year follow-up, a slightly higher rate of all-cause mortality (9.8% vs. 13.7%, HR 1.4 [0.92–2.29], *p* = 0.10) was observed, but there was no difference in the occurrence of strokes (3.6% vs. 3.3%, *p* = 0.87). **Conclusions**: An LAAPd of >38.5 mm was found to be an independent predictor of POAF after coronary artery bypass grafting and resulted in a non-significant tendency towards a worse outcome regarding all-cause mortality in a five-year follow-up.

## 1. Introduction

Postoperative atrial fibrillation (POAF) is one of the most frequent complications accompanying any type of cardiac surgery [1,2], as well as non-cardiac surgery [2,3]. Once it occurs, POAF is associated with decreased cognitive and renal function, longer stays in intensive care and hospitals in general, increased healthcare costs, as well as elevated postoperative mortality [4,5]. POAF is known to lead to persistent atrial fibrillation (AF) and represents the most common form of secondary AF, carrying long-term risks for cardiac decompensation, stroke, and mortality [4,5]. Therefore, the current guidelines recommend frequency control, if possible, restoring and maintaining sinus rhythm with antiarrhythmic medications or electrical cardioversion concomitant to anticoagulation to reduce intracardiac thrombus formation [6,7]. 

However, the pathophysiology of POAF is multifactorial and involves a wide range of risk factors such as age, diabetes, hypertension, obesity, impaired cardiac function, or postoperative complications like pericardial effusion [8,9,10,11]. Age is repeatedly reported as the primary predictor of POAF [12,13]. Another significant factor is the cardiac geometry, particularly regarding left atrial size, since an increased left atrial size is closely associated with a higher likelihood of developing POAF [10,14,15]. Left atrial enlargement has even been shown to be closely linked to nonsurgical AF [16]. Left atrial enlargement is believed to result from or be caused by elevated filling pressures, leading to an up-regulation of the renin-angiotensin-aldosterone system, vascular stiffening, endothelial dysfunction, impaired myocyte relaxation, and eventually, resulting in cell death and fibrosis [17,18,19]. Ultimately, these processes may lead to electrical instability in the stressed and remodeled left atrial walls.

A thorough comprehension of surgery-dependent or patient-dependent (cardiac and non-cardiac) risk factors for POAF is of utmost importance to define risk-dependent thresholds and develop strategies to mitigate any interventions or maneuvers triggering arrhythmia, as well as devising strategies to identify and target patients at risk before, during, and after surgery. However, the current guidelines only provide general recommendations on the use of antiarrhythmic medications such as beta-blockers or amiodarone, without offering further targeted and risk factor-dependent advice [6,7].

The association between the left atrial size and the development of POAF in patients undergoing off-pump coronary artery bypass grafting (OPCAB) is an intriguing area of interest, since this surgical procedure avoids the penetration of cardiac chambers, cardioplegia, and extracorporeal circulation [20]. Additionally, an analysis of the long-term association between left atrial size and all-cause mortality and stroke in a mid- and long-term follow-up analysis has yet to be assessed. Therefore, we conducted a retrospective analysis of patients undergoing OPCAB surgery to detect the association between POAF and the left atrial size, the corresponding cut-off point, and the 5-year survival outcome of these patients.

## 2. Patients and Methods

### 2.1. Ethical Statement

Approval, including a waiver of patient consent, was obtained from the local ethics committee of the Ruhr University Bochum (No: 2020-688_1; Date: 12 August 2022). The study was conducted in accordance with the STrengthening the Reporting of OBservational studies in Epidemiology (STROBE) statement (www.strobe-statement.org, access date: 31 May 2024) and in accordance with the ethical standards outlined in the 1964 Declaration of Helsinki and its later amendments. No funding was received for this research.

### 2.2. Patient Recruitment

The retrospective selection criteria encompassed patients undergoing isolated OPCAB surgery and without a history of atrial fibrillation between January 2014 and December 2016 at the Herz- und Diabeteszentrum NRW (Bad Oeynhausen, Germany). Exclusion criteria were defined as concomitant valvulopathies, pulmonary hypertension, the execution of a left atrial appendage amputation or ligation, and patients with missing data.

### 2.3. Echocardiography

All patients received a preoperative transthoracic echocardiography. The left atrial size was measured using M-Mode, assessing the left atrial anterior–posterior diameter (LAAPd) in the parasternal long axis. Patients with missing data regarding the LAAPd, due to either insufficient echocardiography documentation or challenging parasternal image quality with no measurement of the LAAPd, were defined as missing. 

### 2.4. Primary Outcome

The primary outcome was defined as the occurrence of postoperative atrial fibrillation (POAF). The retrospective identification of the occurrence of POAF was guided by a detailed browsing flowchart previously described [21]. In summary, all reports, including prehospital and discharge records, medication, any custodial or visit documentation, and any other documented occurrences of POAF in the patient data management systems of the standard care (ORBIS, Dedalus HealthCare, Bonn, Germany) and intensive care units (COPRA, COPRA System GmbH; Berlin, Germany), were reviewed for arrhythmias. Furthermore, all electrocardiograms (ECG) were examined for the occurrence of POAF, which were performed at least four times for each patient: on admission, immediately after surgical intervention, on the first postoperative day, and before discharge.

### 2.5. Secondary Outcomes and Follow-up

The secondary outcomes were defined as all-cause mortality and stroke in a 5-year follow-up period. Criteria for reaching the endpoint of stroke included confirmation through cerebral imaging (computed tomography or MRI), the presence of unequivocal neurological impairment, such as hemiplegia, or instances of stroke confirmed by a neurologist. Four sources of information were used during follow-up: a review of our medical records; an annual, standardized form (post-discharge) completed by the patients themselves, as well as by their out-patient care physician and an annual consultation with the respective registration office in case of missing post-discharge forms. Follow-up data were assessed from the date of surgery (2014–2016) until December 2021. Patients were censored at their last follow-up.

### 2.6. Statistical Analysis

Statistical analysis was performed using SPSS software (Version 28, IBM, New York, NY, USA) and R (Version 4.2.2, R Core Team, Vienna, Austria). Categorical variables are presented as absolute and relative frequencies, while continuous variables are presented as means with standard deviations. To detect a correlation between LAAPd and POAF occurrence, a Pearson correlation analysis was conducted, followed by a receiver-operating characteristic (ROC) curve analysis to identify the cut-off point based on the best sensitivity and specificity. An independent risk factor analysis was performed, initially using a univariate logistic regression model for the five most common predictors of POAF. Variables reaching a *p*-value of ≤0.10 were then included in a multivariate logistic regression model, with those variables reaching a *p*-value of <0.05 considered as independent risk factors.

Following the cut-off point detection, the cohort was divided into two groups based on this point. Given the cohort and group definition was chosen in a non-randomized fashion, we conducted 1:1 PS matching using the available baseline characteristics, utilizing nearest neighbor matching with a caliper of 0.2. Sufficient matching was present if the standardized mean difference was <|0.1| [22]. 

Primary endpoint analysis was performed using a χ^2^-test. Secondary outcomes were assessed through a time-to-event analysis concerning the primary composite endpoint and the secondary endpoints with the use of Kaplan–Meier survival curves and a Cox proportional-hazards model. Additionally, a competing risk analysis was performed utilizing the Fine–Gray model to analyze mortality and stroke, which is provided within the Supplemental Data. 

Parameter estimates are presented with their hazard or odds ratio, 95% confidence interval (95%CI), and the corresponding *p*-value. *p*-values < 0.05 were considered statistically significant.

## 3. Results

### 3.1. Study Cohort

**Figure 1:**
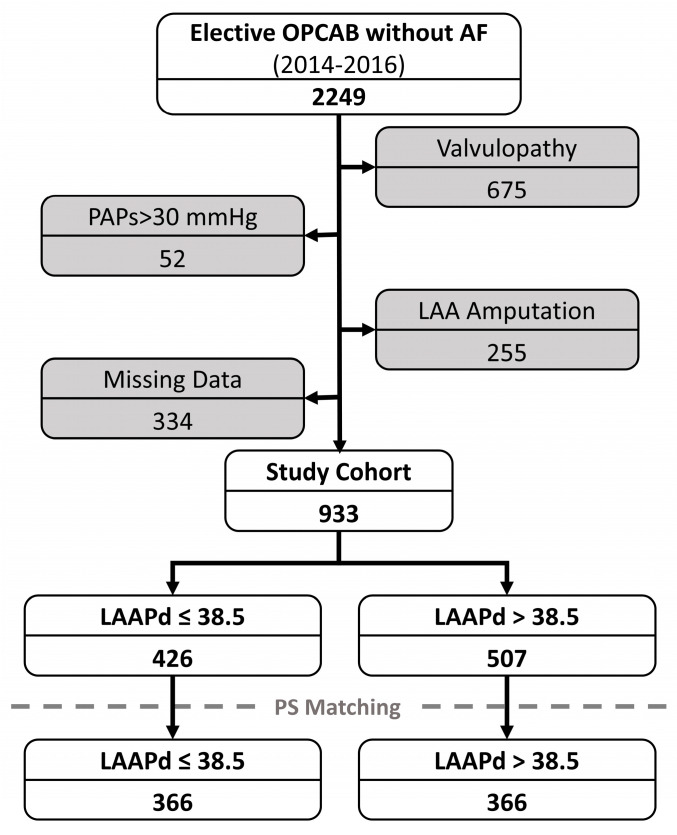
Patient selection process. LAA, left atrial appendage; LAAPd, left atrial anterior–posterior diameter; OPCAB, off-pump coronary artery bypass grafting.

In total, 2249 patients who underwent isolated off-pump coronary artery bypass grafting without a history of AF were identified. Concomitant valvulopathy led to the exclusion of 675 patients, pulmonary hypertension led to the exclusion of 52 patients, left atrial appendage amputation led to the exclusion of 255 patients, and 334 patients were excluded due to missing data, resulting in a final cohort of 933 patients. The baseline characteristics of the total cohort are summarized in Table 1, whereas the patient selection is illustrated in Figure 1. 

### 3.2. ROC Analysis and Pearson Correlation

**Figure 2:**
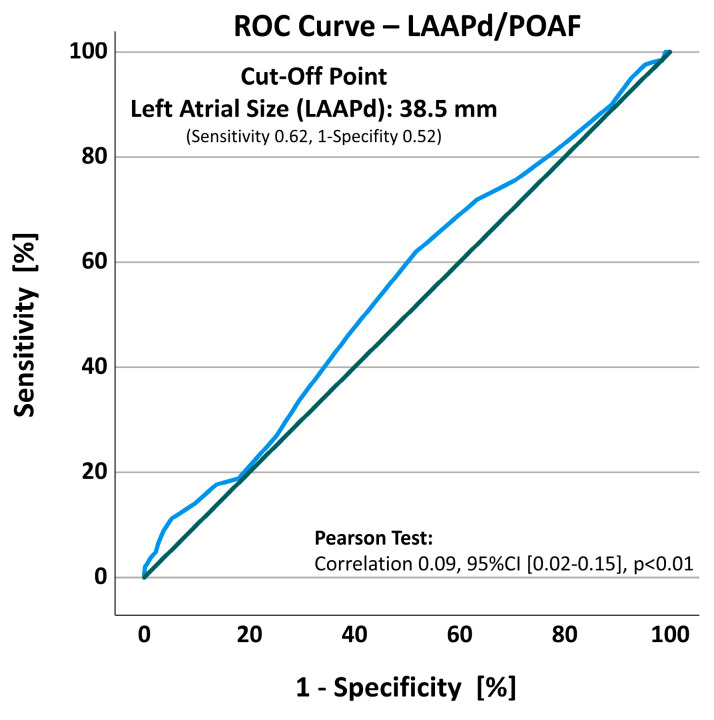
ROC curve (blue) assessing the sensitivity and specificity of left atrial diameter to the occurrence of POAF. Standard line (green). 95%CI, 95% confidence intervals; LAAPd, left atrial anterior–posterior diameter; POAF, postoperative atrial fibrillation; ROC, receiver-operating characteristic.

Pearson correlation assessing the association of the LAAPd to the occurrence of POAF revealed a weak but significant correlation (correlation 0.09, 95%CI [0.02–0.15], *p* < 0.01). The ROC curve analysis revealed a cut-off point at an LAAPd of 38.5 mm with a sensitivity of 0.62 and a 1-specificity of 0.52 (Figure 2). 

### 3.3. Univariate and Multivariate Logistic Regression

Univariate logistic regressions were applied to five criteria that were suspected to be in association with the development of POAF. These were the left atrial size (LAAPd), the BMI, the left ventricular ejection fraction (LVEF), patient’s age, and the CHA_2_DS_2_-VASc-score, which is used to predict stroke risk in patients with AF. Univariate regression showed a significant result for the LAAPd (*p* < 0.01), LVEF (*p* = 0.07), age (*p* < 0.001), and CHA_2_DS_2_-VASc-score (*p* < 0.01). Multivariate logistic regression confirmed the LAAPd (*p* < 0.02) and age (*p* = 0.01) to be independent risk factors. The results of the uni- and multivariate regression are illustrated in Table 1.

### 3.4. Cohorts with Preserved and Enlarged LA Diameter

After identifying an LAAPd of 38.5 mm, the study cohort was divided into two groups: those with a preserved LA size (LA diameter ≤ 38.5 mm) and those with an enlarged LA (LA diameter > 38.5 mm), encompassing 426 and 507 patients, respectively. Baseline characteristics of the unmatched cohorts revealed 11 parameters with an SMD > |0.1|. Following propensity score matching, the final study cohort consisted of 933 patients with 366 patients in each group, achieving sufficient group comparability with no baseline variable exceeding an SMD of |0.1|. Baseline information for the unmatched and matched cohorts is summarized in Table 2. 

### 3.5. Incidence of POAF

**Figure 3:**
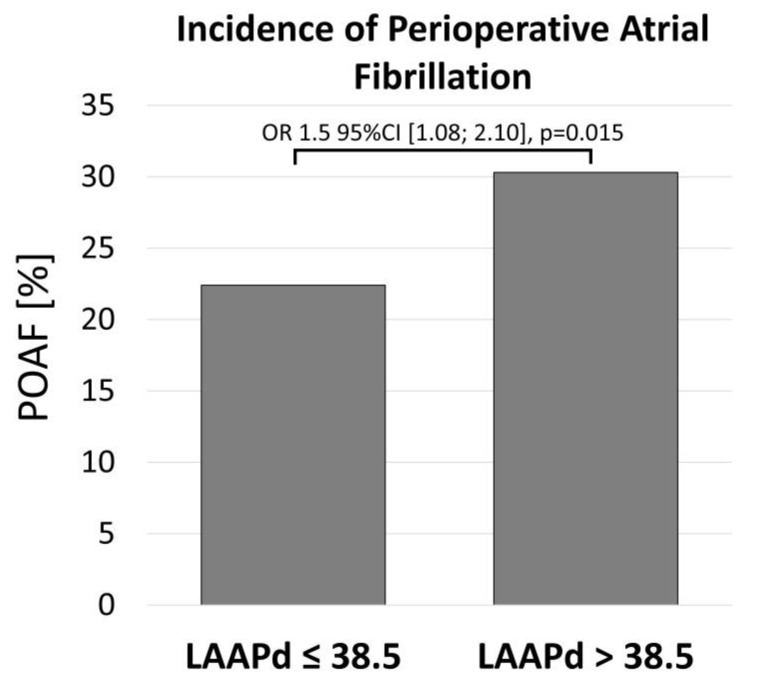
Incidence of postoperative atrial fibrillation in patients with preserved and enlarged left atrial diameter with a cut-off of 38.5 mm. 95%CI, 95% confidence intervals; LAAPd, left atrial anterior–posterior diameter; OR, odds ratio; POAF, postoperative atrial fibrillation.

Primary outcome analysis regarding the occurrence of POAF showed a significantly higher rate of POAF in patients with enlarged LA diameter, with 22.4% vs. 30.3% (OR 1.5, 95%CI [1.08–2.01], *p* = 0.015) in patients with an LAAPd_≤38.5_ and LAAPd_>38.5_, respectively (Table 3, Figure 3). Unmatched results are provided in Supplementary Table S1 and Supplementary Figure S1.

### 3.6. All-Cause Mortality and Stroke in a 5-Year Follow-up

**Figure 4:**
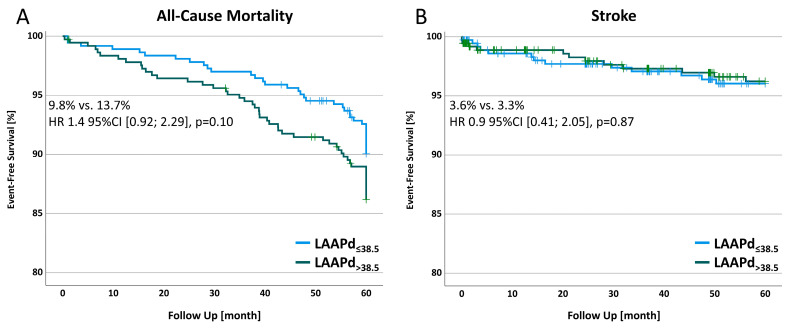
All-cause mortality (**A**) and stroke (**B**) in a 5-year follow-up. 95%CI, 95% confidence intervals; HR, hazard ratio; LAAPd, left atrial anterior–posterior diameter.

Five-year results indicated a tendency towards higher all-cause mortality in patients with an enlarged left atrium with 9.8% vs. 13.7% (OR 1.4 [0.92–2.29], *p* = 0.10); however, this difference did not show statistical significance. Regarding the stroke rate, similar results were observed in both groups with 3.6% and 3.3% (OR 0.9 95%CI [0.41–3.05], *p* = 0.87). Detailed results and survival curves are provided in Table 3 and Figure 4. Unmatched results are provided in Supplementary Table S1 and Supplementary Figure S2. These findings were in concordance with the competing risk analysis (all-cause mortality (*p* = 0.10), stroke (*p* = 0.82), Supplementary Table S2).

## 4. Discussion

This study emphasizes the association between left atrial geometry and the risk of developing postoperative atrial fibrillation. The main findings of the analysis are four-fold: The left atrial anterior–posterior diameter (LAAPd) is an independent risk factor for the occurrence of POAF in patients undergoing coronary artery bypass grafting (I), The incidence of POAF increases with an increasing LAAPd with a cut-off point at 38.5 mm (II), patients with an LAAPd of >38.5 show a higher rate of POAF (III) and a tendency towards higher all-cause mortality (IV). 

The current analysis demonstrates a weak but significant association of LAAPd to the development of POAF in the ROC and Pearson correlation (*p* < 0.01), while the cut-off point of the LAAPd was found to be 38.5 mm (sensitivity 0.62, 1-specifity 0.52, Figure 2), which is in concordance with similar findings of Karimi et al. [23]. Despite the weak correlation, LAAPd was the only independent risk factor in both univariate (*p* < 0.01) and multivariate analyses (*p* = 0.02), in addition to age. These results align with observations of different researchers assessing risk factors for POAF development, although some studies present other factors such as the body mass index (BMI) [9,24], acute myocardial infarction [25], or pericardial effusion [11] to be predictive of POAF occurrence. Contrary results in this regard highlight the multifactorial origin of AF and POAF, raising questions about differences in the examined cohorts or surgical procedures. However, age and left atrial size remain the most repeatedly identified predictors of POAF [12,26]. Other than POAF development, age is also known to predispose individuals to a higher stroke rate and all-cause mortality, resulting in age being a major part of any scoring system, e.g., the CHA_2_DS_2_-VASc-score [27].

In terms of left atrial size, there are several standards to use for measurement [6,14]. However, the left atrial volume index (LAVI) has proven superior in describing the left atrial size relative to patient size [28,29]. In the search for a cut-off point, Osranek et. al, like others, identified an LAVI of >32 mL/m^2^ to predict the occurrence of POAF [10,30]. Nonetheless, the debate regarding the most predictive and pragmatic parameter of the left atrial size is still ongoing between LAAPd, LAV, and LAVI. 

The left atrial anterior–posterior diameter (LAAPd) measured by M-Mode in the parasternal long axis, presents the simplest and most commonly used method for measuring left atrial size and is often the first parameter taken for the left atrium [14]. Despite its inferiority in accuracy in describing the left atrial size compared to LAV or LAVI [14,28], our results demonstrate that the LAAPd still exhibits a significant correlation with the development of POAF as an independent risk factor, which could also be observed in other studies [31,32]. The calculated cut-off point of 38.5 mm is slightly below the maximal reference value of 40 mm [14]. Zacà et al. described 40 mm as the cut-off point in their analysis regarding the association of LAAPd and POAF [33]. The observed data, which show statistical significance with a weak correlation, have to be proven regarding their clinical significance by further assessments of the left atrium. However, since the LAAPd is sometimes the only measurement to interpret LA size, a value above 38.5 mm could be used to recommend further measurements such as LAVI to allow a more comprehensive evaluation of the left atrium in patients at risk across more medical centers. 

All these considerations are of significant interest, as the recognition of independent risk factors is merely the first step, lacking value if not linked to any kind of action aiming to protect the adverse outcome. Regarding POAF, several medical and surgical strategies have been developed, however, a targeted recommendation within the current guidelines remains absent. In terms of medical strategies, beta-blockers (or amiodarone) are recommended for all patients undergoing cardiac surgery [6,7]. Beta blockers, bi-atrial pacing, or intravenous amiodarone have shown POAF reduction [34]. Nevertheless, the PAPABEAR study, assessing preoperative amiodarone usage, failed to present any differences in postoperative complications or in-hospital (6 month, 1 year) mortality, despite the reduction of POAF [35]. Gaudino et al. were able to prove that posterior left pericardiotomy is protective against POAF development [36], which could be further guided by risk stratification through left atrial size assessment. 

Given the strong connection between the pathophysiology of atrial fibrillation and left atrial enlargement, which leads to electrical instability within the left atrial branch path, the role of surgical ablation, a procedure recommended in patients with atrial fibrillation (class IIA/2a) [6,7], may offer advantages. Another surgical strategy of interest is the left atrial appendage (LAA) amputation, which has been proven significantly superior in stroke reduction in patients with atrial fibrillation and an elevated CHA_2_DS_2_-VASc-score [37] and is consequently recommended in patients with AF (class IIA) [7,38]. A benefit of LAA amputation in patients with no history of atrial fibrillation and even similar results in patients with POAF and LAA amputation to patients with maintained SR [21,39] have been demonstrated, while the results of the ongoing LeAAPS trial (NCT05478304) will be of high interest to phrase recommendations for LAA amputation in patients with no history of AF. These recommendations will have to rely on additional risk parameters such as the CHA_2_DS_2_-VASc-score; however, the left atrial size may contain crucial information for decision making. Nonetheless, the hypotheses regarding the protective and predictive associations of the mentioned procedures and strategies require further validation in controlled randomized trials.

Besides the association between the left atrial size and the development of POAF, we investigated the relationship between the LAAPd and a 5-year survival outcome of all-cause mortality and stroke. To mitigate any bias due to group differences caused by the retrospective non-randomized patient selection, propensity score matching was applied to cohorts divided by the calculated cut-off point of an LAAPd of 38.5 mm. Our results revealed a tendency towards higher all-cause mortality with 9.8% vs. 13.7% (OR 1.4 [0.92–2.29], *p* = 0.10), prompting the question of statistical underpowerment and highlighting the hypothesis of risk prediction by left atrial geometry. Although the association of POAF to long-term mortality has already been demonstrated in several studies [40,41], the association of LAAPd to all-cause mortality is weaker despite its prediction of POAF occurrence. The left atrial size is closely linked to diastolic dysfunction and is considered to be an indicator of cardiovascular disease [42]. However, Osranek et al. demonstrated the left atrial size to predict a worse outcome even in non-surgical patients [43], which may be observed in our results as well. The fact that surgery was performed might serve as misleading evidence and warrants further investigation.

Regarding the stroke rate, no difference was observed in patients with an enlarged LA, similar to the results of patients with POAF. These findings may align with several studies regarding POAF indicating no association between POAF and stroke [21,44]. However, conflicting results emerge from short-, mid-, and long-term follow-up studies on the relationship between POAF and stroke [4,45] and for the association between LA size and stroke, only limited data are available. Nevertheless, similar to the data on POAF, other confounding factors that may contribute to these contradictory results need to be investigated to better understand the role of the LA size—as cause or consequence—in the cardiac machinery.

## 5. Limitations

Several limitations apply to our study. First and foremost, our study is limited due to its retrospective and single-center design. Furthermore, although POAF was observed using a dedicated method, smaller, unnoticed asymptomatic arrhythmic events cannot be ruled out. Operative parameters or the use of inotropic drugs that may have impacted the occurrence of POAF were not part of the analysis. Regarding the 5-year analysis, there was no follow-up of rhythm or medication, e.g., anticoagulation. A comparison to the indexed left atrial volume (LAVI), the probably most validated parameter regarding the LA size, was not possible due to missing data. 

## 6. Conclusions

The left atrial anterior–posterior diameter, with a cut-off point at 38.5 mm, was found to be an independent predictor of the occurrence of POAF after coronary artery bypass grafting and resulted in a tendency towards a worse outcome regarding all-cause mortality in a five-year follow-up. 

## Figures and Tables

**Table 1:** Univariate and multivariate logistic regression of parameters in association with the occurrence of POAF.

Variable	*p*-Value
**Univariate Logistic Regression**	
Left atrial size (LAAPd)	**<0.01**
BMI	0.19
LVEF	**0.07**
Age	**<0.001**
CHA_2_DS_2_-VASc-score	**<0.01**
**Multivariate Logistic Regression**	
Left atrial diameter	**0.02**
LVEF	0.11
Age	**0.01**
CHA_2_DS_2_-VASc-score	0.53

Abbreviations: BMI, body mass index; LVEF, left ventricular ejection fraction; CHA_2_DS_2_-VASc-score, congestive heart failure, hypertension, age > 75 (doubled), diabetes, stroke (doubled), vascular disease, age 65 to 74 and sex category (female); LAAPd, left atrial anterior–posterior diameter. Significant results are highlighted in bold font.

**Table 2:** Baseline characteristics of the total cohort, the unmatched and the propensity score matched cohorts.

Variable	All	Unmatched Cohorts					Propensity Score Matched Cohorts				
		LAAPd_≤38.5 mm_ n (%)	LAAPd_>38.5 mm_ n (%)	OR [95%CI]	SMD	*p*-Value	LAAPd_≤38.5 mm_ n (%)	LAAPd_>38.5 mm_ n (%)	OR [95%CI]	SMD	*p*-Value
	933 (100)	426 (100)	507 (100)				366 (100.0)	366 (100.0)			
Preop. MI	256 (27.4)	121 (28.4)	135 (26.6)	0.9 [0.69–1.22]	−0.040	0.55	104 (28.4)	103 (28.1)	1.0 [0.72–1.36]	−0.006	0.94
AHT	801 (85.9)	353 (82.9)	448 (88.4)	1.6 [1.08–2.27]	0.172	**0.016**	305 (83.3)	314 (85.8)	1.2 [0.81–1.81]	0.077	0.36
Smoking	448 (48)	210 (49.3)	238 (47.0)	0.9 [0.70–1.18]	−0.047	0.47	180 (49.2)	185 (50.6)	1.1 [0.79–1.41]	0.027	0.71
Diabetes	309 (33.1)	138 (32.4)	171 (33.7)	1.1 [0.81–1.40]	0.028	0.67	116 (31.7)	129 (35.3)	1.2 [0.86–1.60]	0.075	0.31
HLP	863 (92.5)	385 (90.4)	478 (94.3)	1.8 [1.07–2.88]	0.168	**0.024**	337 (92.1)	339 (92.6)	1.1 [0.63–1.86]	0.024	0.78
DVT	13 (1.4)	7 (1.6)	6 (1.2)	0.7 [0.24–2.15]	−0.043	0.55	6 (1.6)	3 (0.8)	0.5 [0.12–2.00]	−0.076	0.31
Preop. Dialysis	6 (0.6)	1 (0.2)	5 (1.00)	4.2 [0.49–36.37]	0.076	0.15	1 (0.3)	3 (0.8)	3.0 [0.30–29.10]	0.055	0.32
COPD	56 (6)	20 (4.7)	36 (7.)	1.6 [0.88–2.72]	0.094	0.12	20 (5.5)	19 (5.2)	1.0 [0.50–1.81]	−0.011	0.87
Beta-blockers	619 (66.3)	278 (65.3)	341 (67.3)	1.1 [0.83–1.44]	0.043	0.52	238 (65.0)	238 (65.0)	1.0 [0.74–1.36]	0.000	>0.99
Ca antagonists	186 (19.9)	69 (16.2)	117 (23.1)	1.6 [1.12–2.16]	0.163	**<0.01**	61 (16.7)	71 (19.4)	1.2 [0.82–1.76]	0.065	0.34
Sex [female]	160 (17.1)	87 (20.4)	73 (14.4)	0.7 [0.47–0.92]	−0.171	**0.015**	62 (16.9)	61 (16.7)	1.0 [0.67–1.44]	−0.008	0.92
ASA [class]	2.9 ± 0.5	2.9 ± 0.5	2.9 ± 0.5	−0.11–0.02	0.089	0.21	2.9 ± 0.5	2.9 ± 0.5	−0.11–0.04	0.071	0.39
NYHA [class]	2.0 ± 0.9	2.1 ± 0.9	2.03 ± 0.9	−0.09–0.13	−0.030	0.65	2.0 ± 0.9	2.0 ± 0.9	−0.12–0.12	−0.003	0.97
CCS [class]	2.6 ± 1.2	2.7 ± 1.2	2.5 ± 1.2	0.01–0.32	−0.145	**0.034**	2.6 ± 1.2	2.5 ± 1.2	−0.10–0.23	−0.061	0.41
LVEF [%]	59.1 ± 9.1	60.0 ± 9.0	58.4 ± 9.2	0.43–2.78	−0.176	**<0.01**	59.4 ± 8.7	59.0 ± 9.2	−0.82–1.64	−0.045	0.51
EurosScore II [%]	1.5 ± 1.5	1.6 ± 1.8	1.4 ± 1.2	−0.04–0.34	−0.126	0.12	1.5 ± 1.6	1.5 ± 1.3	−0.24–0.18	0.017	0.84
CHA_2_DS_2_VASc-score [points]	2.4 ± 1.3	2.3 ± 1.3	2.4 ± 1.3	−0.26–0.08	0.064	0.33	2.3 ± 1.3	2.4 ± 1.3	−0.29–0.08	0.079	0.27
Age [years]	64.5 ± 9.4	64.3 ± 9.5	64.6 ± 9.3	−1.59–0.84	0.040	0.55	64.1 ± 9.5	64.4 ± 9.2	−1.71–1.04	0.036	0.64
BMI [kg/m^2^BSA]	28.7 ± 4.2	27.9 ± 4.0	29.4 ± 4.3	−2.07–−0.99	0.354	**<0.01**	28.4 ± 4.0	28.8 ± 4.2	−0.88–0.10	0.090	0.12
TSH [µU/mL]	1.4 ± 2.2	1.3 ± 1.1	1.5 ± 2.8	−0.55–0.02	0.091	0.08	1.3 ± 1.1	1.3 ± 1.3	−0.17–0.16	<0.001	>0.99
Potassium [mmol/L]	3.9 ± 0.8	3.8 ± 0.9	3.9 ± 0.7	−0.21–0.00	0.142	0.06	3.9 ± 0.8	3.9 ± 0.7	−0.16–0.04	0.079	0.27
Sodium [mmol/L]	135.2 ± 24.4	133.6 ± 28.2	136.6 ± 20.5	−6.14–0.14	0.146	0.07	135.3 ± 24.0	136.6 ± 20.6	−4.20–1.68	0.061	0.40
Calcium [mmol/L]	2.3 ± 0.4	2.3 ± 0.5	2.4 ± 0.4	−0.11–0.00	0.145	0.07	2.3 ± 0.4	2.4 ± 0.4	−0.08–0.02	0.068	0.36
Creatinine [mg/dL]	1.0 ± 0.5	1.0 ± 0.3	1.1 ± 0.6	−0.15–−0.02	0.132	**0.014**	1.0 ± 0.3	1.0 ± 0.5	−0.10–0.02	0.064	0.22

Abbreviations: 95%CI, 95% confidence intervals; AHT, arterial hypertension; ASA, American Society of Anesthesiologists; BMI, body mass index; Ca, calcium; CCS, Canadian Cardiovascular Society classification; CHA2DS2VASc-score, congestive heart failure, hypertension, age >75 (doubled), diabetes, stroke (doubled), vascular disease, age 65 to 74 and sex category (female); COPD, chronic obstructive pulmonary disease; DVT, deep vein thrombosis; HLP, hyperlipidemia; LAAPd, left atrial anterior–posterior diameter; LVEF, left ventricular ejection fraction; NYHA, New York Heart Association classification; OR, odds ratio; Preop., preoperative; TSH, thyroid-stimulating hormone. Significant results are highlighted in bold font.

**Table 3:** Outcome of POAF and 5-year follow-up of all-cause mortality and stroke in patients with preserved and enlarged left atrial size (LAAPd).

Variable	LAAPd ≤ 38.5 mm n (%)	LAAPd > 38.5 mm n (%)	OR/HR [95%CI]	*p*-Value
	366 (100)	366 (100)		
**Primary Endpoint**				
**POAF**	82 (22.4)	111 (30.3)	1.5 [1.08–2.10]	0.015
**Secondary End-Point (5y Follow-Up)**				
All-cause mortality	36 (9.8)	50 (13.7)	1.4 [0.92–2.29]	0.10
Stroke	13 (3.6)	12 (3.3)	0.9 [0.41–2.05]	0.87

Abbreviations: 95%CI, 95% confidence intervals; HR, hazard ratio; LAAPd, Left atrial anterior–posterior diameter; OR, odds ratio; POAF, postoperative atrial fibrillation. Significant results are highlighted in bold font.

## Data Availability

The data underlying this article will be shared upon reasonable request to the corresponding author.

## Supplementary Materials

The following supporting information can be downloaded at: https://www.mdpi.com/article/10.3390/jcm13133738/s1, Figure S1: Incidence of perioperative atrial fibrillation in patients with preserved and enlarged left atrial diameter with a cut-off of 38.5 mm in the unmatched cohort; Figure S2: All-cause mortality and stroke in a 5-year follow up in the unmatched cohort; Table S1: Outcome of POAF and 5-year follow up of all-cause mortality and stroke in patients with preserved and enlarged left atrial diameter in the unmatched cohort; Table S2: Competing risk analysis (Fine-Gray model) of the 5-year follow up of all-cause mortality and stroke in patients with preserved and enlarged left atrial diameter in the unmatched cohort.

## Abbreviations

95%CI	95% confidence interval
AF	Atrial fibrillation
AHT	Arterial hypertension
ASA	American Society of Anesthesiologists
BMI	Body mass index
Ca	Calcium
CAD	Coronary artery disease
CCS	Canadian Cardiovascular Society
CHA_2_DS_2_-VASc	congestive heart failure, hypertension, age >75 (doubled), diabetes, stroke (doubled), vascular disease, age 65 to 74 and sex category (female)
COPD	Chronic obstructive pulmonary disease
CRT	Controlled randomized trial
DVT	Deep vein thrombosis
ECLS	Extracorporeal life support
LA	Left atrium
LAA	Left atrial appendage
LVEF	Left ventricular ejection fraction
HLP	Hyperlipidemia
HR	Hazard ratio
IABP	Intra-aortic balloon pump
NYHA	New York Heart Association
OPCAB	Off-pump coronary artery bypass grafting
OR	Odds Ratio
POAF	Postoperative atrial fibrillation
Preop.	Preoperative
PS	Propensity Score
ROC	Receiver operating characteristic
SD	Standard deviation
SMD	Standardized mean difference
SR	Sinus rhythm
STROBE	STrengthening the Reporting of OBservational studies in Epidemiology
TSH	Thyroid-stimulating hormone
