# Supplementary material for: Postoperative Atrial Fibrillation Prediction by Left Atrial Size in Coronary Artery Bypass Grafting and Five-Year Survival Outcome
Source: J Clin Med. 2024 Jun 26;13(13):3738. doi: 10.3390/jcm13133738 (PMC11242862; doi:10.3390/jcm13133738)
Supplement: Supplementary file 1 [file jcm-13-03738-s001.zip › jcm-3061807-supplementary.pdf]

Table S1. Outcome of POAF and 5-year follow up of all-cause mortality and stroke in patients with preserved and enlarged left atrial diameter in the unmatched cohort.

Variable	LAAPd ≤ 38.5 mm n (%)	LAAPd > 38.5 mm n (%)	OR/HR [95%CI]	p-value
	426 (100)	507 (100)		
**Primary Endpoint**				
POAF	95 (22.3)	154 (30.4)	1.5 [1.13-2.04]	<0.01
**Secondary End-Point (5y Follow Up)**				
All-Cause Mortality	39 (9.2)	66 (13.0)	1.5 [0.98-2.26]	0.06
Stroke	15 (3.5)	16 (3.2)	0.9 [0.44-1.83]	0.78

Abbreviations: 95%CI, 95% Confidence intervals; HR, Hazard ratio; LAAPd, Left atrial anterior-posterior diameter; OR, Odds ratio; POAF, Perioperative atrial fibrillation.

Table S2. Competing risk analysis (Fine-Gray model) of the 5-year follow up of all-cause mortality and stroke in patients with preserved and enlarged left atrial diameter in the unmatched cohort.

Variable	p-value unmatched	p-value PS matched
All-Cause Mortality	0.06	0.10
Stroke	0.82	0.82

Supplementary Figures

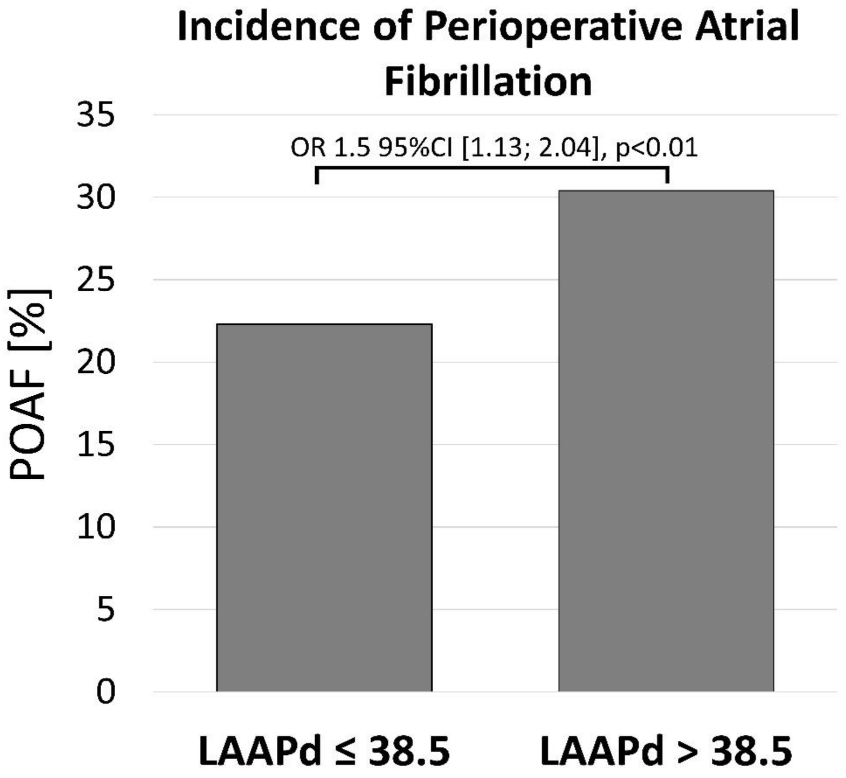

Figure S1. Incidence of perioperative atrial fibrillation in patients with preserved and enlarged left atrial diameter with a cut-off of 38.5 mm in the unmatched Cohort. 95%CI, 95% Confidence intervals; LAAPd, Left atrial anterior-posterior diameter; OR, Odds ratio; POAF, Perioperative atrial fibrillation.

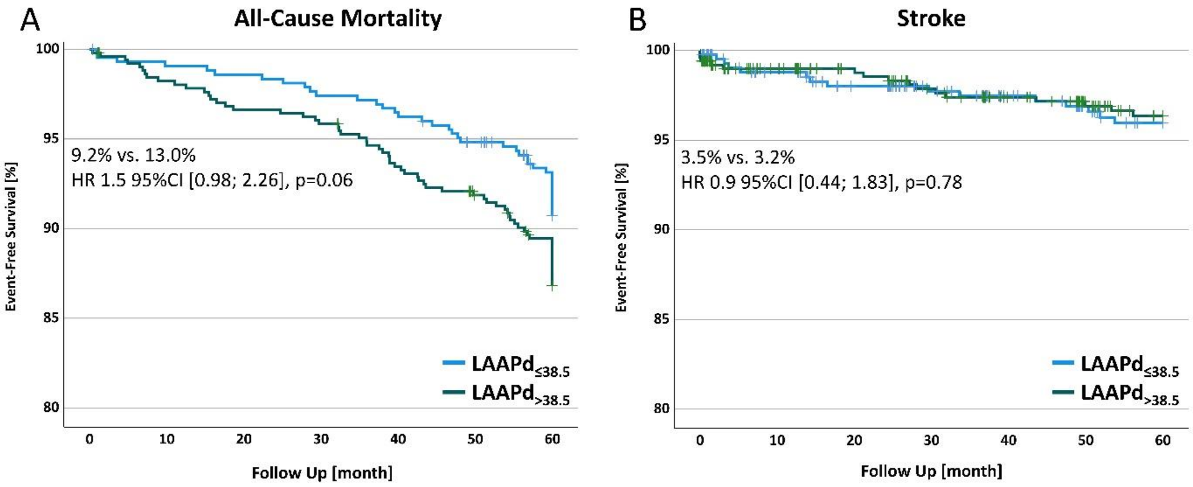

Figure S2. All-cause mortality and stroke in a 5-year follow up in the unmatched cohort. 95%CI, 95% Confidence intervals; HR, Hazard ratio; LAAPd, Left atrial anterior-posterior diameter.
